# ‘Expansion *in-situ*’ concept as a new technique for expanding skin and soft tissue

**DOI:** 10.3892/etm.2013.1269

**Published:** 2013-08-22

**Authors:** LIN FANG, CHUANDE ZHOU, MINGYONG YANG

**Affiliations:** Department of Microinvasive Plastic Surgery, Plastic Surgery Hospital, Peking Union Medical College and Chinese Academy of Medical Sciences, Beijing 100144, P.R. China

**Keywords:** soft tissue expansion, incision dehiscence, expansion *in-situ*, additional incision, cicatrix

## Abstract

Techniques for expanding skin and soft tissue are widely used to repair damaged areas since they facilitate the provision of new, additional skin tissue with similar quality, texture and color to that surrounding the defective area. Conventional expansion techniques involve placing expanders under the normal skin adjacent to a lesion. However, these techniques may involve additional incisions, complications with blood supply and ‘dog-ear’ deformities and may result in a low utilization rate of the expanded tissue. When reconstructing small defects that may not be sutured directly, these shortcomings, particularly the requirement to make additional incisions, limit the application of conventional techniques. The current study presents a novel approach to expansion called the ‘expansion *in-situ*’ technique. In this technique, the lesion is used as the center for expansion and expanders of optimal size are implanted under the lesion and surrounding normal soft tissue. Following expansion, the damaged area is excised directly. In order to avoid poor healing of the incision made during expander implantation, the overlapping suturing of both cut sides is conducted. This enlarges the contact area of both sides of the incision, thereby avoiding incision dehiscence and increasing wound healing during the expansion process. Between August 2006 and July 2011, the expansion *in-situ* technique was applied in 10 cases involving either nevus excision or scar removal. All 10 cases were treated successfully. Five of the cases were followed up over 1–3 years. The ‘expansion *in-situ*’ technique is likely to be useful for avoiding additional incisions and improving the utilization rate of expanded skin flaps.

## Introduction

In order to reconstruct small- or medium-sized soft tissue defects following lesion excision, there are a number of operative approaches available to plastic surgeons. These include skin graft, local flap and free-tissue transfer procedures (1–3). These techniques often make use of tissues from a donor site to meet the reconstructive requirements of the recipient site. Soft-tissue expansion techniques generate extra skin tissue from donor sites adjacent to the lesion with similar characteristics to the damaged skin; for example, appropriate color match, fine texture, sensibility and substantial adnexa to aesthetically restore the resulting defect. To obtain extra skin tissue via soft-tissue expansion, one or more temporary expanders are implanted under the skin. The expanders are capable of accumulating saline solution via injection through a gel-filled valve system in the reservoir dome. The internal pressure of the expander exerts force on the soft tissue of the donor site, which gradually expands, providing additional tissue for the recipient area while the donor site remains preserved. Temporary expanders are available in different sizes and shapes, depending on the clinical reconstructive requirements. This expansion technique safely and effectively enhances methods used in plastic surgery. Plastic surgeons have a number of common preferences regarding the placement positions and levels of soft-tissue expanders, and have also investigated methods to prevent complications of this technique (4–6). Models for estimating the expanded soft tissue during the expansion process have been created (7,8). However, certain inevitable consequences, including visible scars and, in particular, additional incision scars, dissatisfy patients. Although additional incisions are unavoidable when unfurling hemispheric expanded flaps and correcting ‘dog-ear’ deformities, reducing the scar burden as much as possible via the use of expanded flaps is essential, particularly in exposed areas, including the face and neck. In addition, shortening the treatment time to reduce the cost of treatment is also important. The shortcomings of the tissue expansion technique, including the production of additional incision scars, prolonged expansion time and high rates of complication, limit its use in the treatment of small defects of the face, neck and limbs that may not be excised and sutured directly in one procedure. In order to solve these problems, certain improvements have been gradually applied and achieved during clinical experience. In the present study, tissue expanders were implanted under the lesions, using the lesions as the center and fully expanding the surrounding normal tissue, so as to reduce the relative size of the lesions. This method has been named the ‘expansion *in-situ*’ technique. It not only reduces the length of additional incisions and the number of tissue expanders used, but it also shortens the expansion period. The expanded skin tissue surrounds the lesion in all directions and is fully utilized so that closure of the defect by direct suturing may be performed without tension. In the present study, the expansion *in-situ* technique was applied in 10 cases to repair small- and medium-sized soft-tissue defects, which it was not possible to suture directly. Successful results were achieved within a shorter period of time than traditional expansion techniques.

## Materials and methods

### 

#### Patients

Between August 2006 and December 2011, the expansion *in-situ* technique was applied to 10 patients (4 females and 6 males) in order to reconstruct soft tissue defects resulting from nevi (n=5) and scar excisions (n=5) located on the face and upper limbs. The largest defect was 15×7.5 cm, resulting from a facial scar excision. The smallest defect was 3×2.5 cm, resulting from removal of a nevus on the limb. The remaining 8 cases comprised of 4 cases of cicatrices (2 face and 2 upper limb) and 4 cases of nevi (1 face and 3 upper limb). The age range of the patients was 12–30 years (mean, 21.3 years). The volume of the tissue expanders was 50–400 ml depending on the size and characteristics of the defect. The duration of tissue expander inflation ranged from 6 to 10 weeks (mean, 8 weeks; Table I). This study was conducted in accordance with the Declaration of Helsinki and with approval from the Ethics Committee of the Chinese Academy of Medical Sciences (Beijing, China). Written informed consent was obtained from all participants.

#### Surgical techniques

The first stage of the surgery involved the insertion of a tissue expander and its serial expansion. The operative design used the lesion as the center mark for a rhombic incision line, the length and width of which did not exceed the size of the lesion. With the patient under anesthesia, the superficial tissue in the marked rhombic area was excised. A pocket was created subcutaneously or to the level of the deep fascia. It was necessary to ensure good hemostasis in the expander pocket. An optimally sized rectangular tissue expander was placed into the pocket and a drainage tube was placed beneath the expander simultaneously. The injection port was placed so as to facilitate inflation of the tissue expander. Overlapping suturing of the de-epithelialized dermal flaps on both sides of the incision was conducted. A pressure dressing was applied to the operative area. From 10 days after surgery, the expander was serially inflated with saline solution on a weekly basis until an adequate volume was achieved. The expander was removed in the second stage of the surgery when an adequate volume was achieved and sufficient stable skin had been generated. The lesion was excised and the incision was sutured directly during this surgery. If the incision was too long, an ‘S-shaped’ suture was created. A drainage tube was placed intraoperatively and removed 3–5 days after the operation. Following a day of observation, the patient was able to be discharged. The suture was removed at 10–12 days postoperatively.

## Results

A total of 10 cases were completed, including complete nevus resection and scar removal. The average time required for tissue expansion was 8 weeks. Necrosis of the skin flaps did not occur. All wounds were closed directly, with the exception of one case of poor wound healing. In this case, the wound finally healed well after a careful dressing change. There were no cases of infection, wound dehiscence or other complications. A follow-up survey was returned by five respondents who agreed to attend a check-up. The average follow-up period was 19.2 months. No hyperplasia of incision scars or relapse of lesions was reported and all the patients who were followed up were satisfied with their final reconstructive and aesthetic outcomes.

### Typical cases

#### Case 1

**Figure 1.**
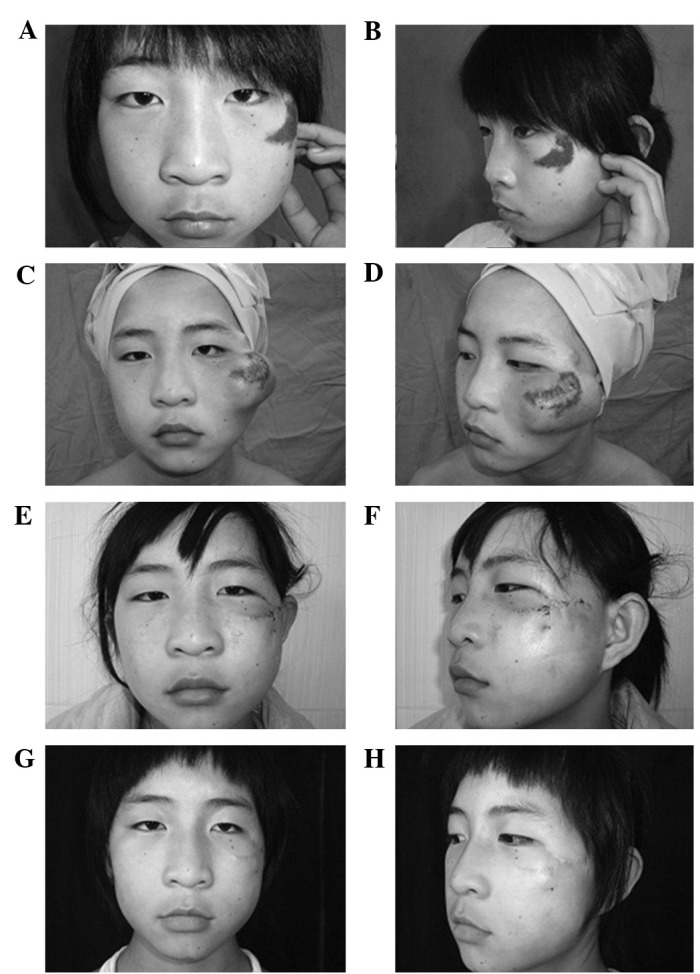
Case 1. Facial giant nevus excision with expansion *in-situ* technique. (A and B) Preoperative frontal and oblique view of a 16-year-old female patient presented with a giant nevus. (C and D) The front and oblique view after 7-week expansion *in-situ*. (E and F) The early postoperative front and oblique view. (G and H) The front and oblique view one year after the operation.

A 16-year-old female patient presented with a giant nevus. The conventional approach to treatment would be the implantation of an expander in the buccal or temporal regions. This approach is likely to have involved excessively long additional incisions and visible ‘dog-ear’ deformities. An alternative approach is the use of an expanded forehead flap based on the superficial temporal vessel or an expanded medial arm flap. However, these approaches would take a long time and may have led to obvious donor site morbidities. Instead, a 50-ml rectangular tissue expander was implanted under the nevus and its circumambient normal skin. Partial epidermal removal of the nevus and overlapping suturing of the remaining dermal flaps was conducted to ensure smooth healing of the incision. After 7 weeks of inflation with saline, the nevus was excised with the 3.0×2.5 cm defect and the expanded flaps were transferred and sutured directly without additional incisions. After one year of follow-up, the patient was satisfied with the results (Fig. 1).

#### Case 3

**Figure 2.**
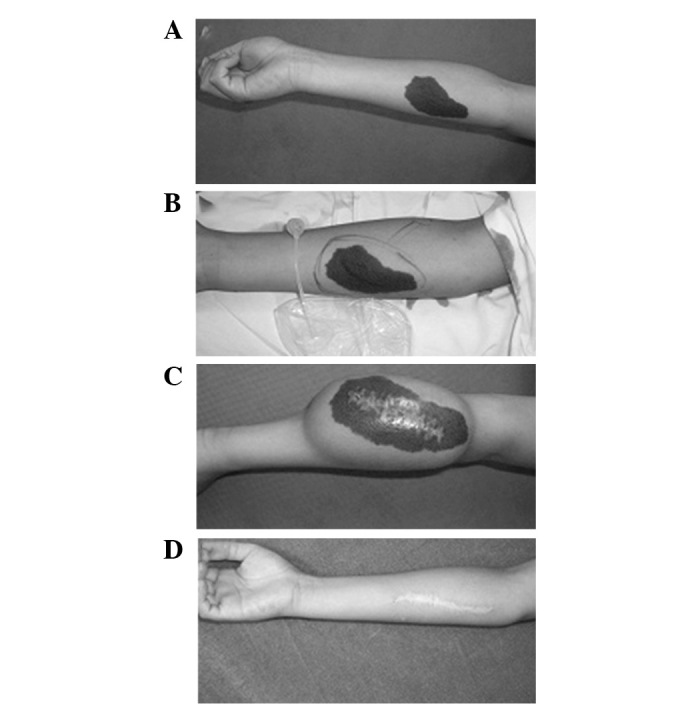
Case 3. Removal of nevus in the limbs with expansion *in-situ* technique. (A) Preoperative view of a 12-year-old boy presented with congenital nevus in right forearm. (B) Design of location of expander; (C) view after the expansion in-situ; (D) appearance one year after surgery.

A 12-year-old male presented with a congenital nevus on the right forearm. The nevus was 10.0×6.0 cm in size. Reconstruction using an autologous skin graft to repair the defect after nevus excision may have led to a difference in the texture and color of the skin in the recipient area. The patient also was unable to accept resection more than once. Traditional expansion techniques would have involved placing expanders under both sides of the nevus. These approaches are of low efficiency since it is not possible to apply the expanded flap effectively in the transfer process. The resulting wound with additional incision also discomforted. Therefore, the incision was performed inside the nevus and part of the nevus was excised. Overlapping suturing of the dermal tissue was conducted in order to ensure smooth healing of the incision. A 300 ml rectangular tissue expander was implanted under the nevus and surrounding normal skin area. The expander was inflated for 10 weeks. In a second-stage procedure, the nevus was resected completely with only a short incision. One year later, there was no relapse and the scar was acceptable (Fig. 2).

#### Case 5

**Figure 3.**
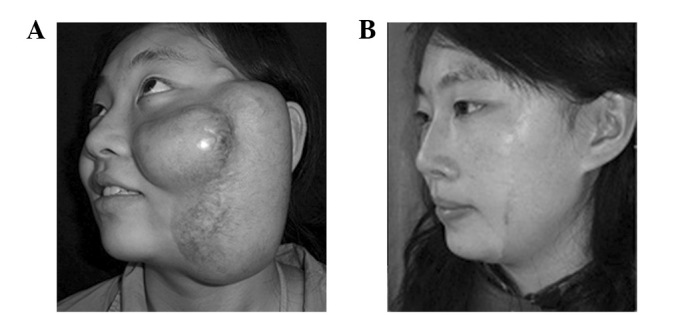
Case 5. Excision of facial scar with the expansion *in-situ* technique. (A) Post-expansion view of a 17-year-old female who presented with a left facial rectangular scar. (B) The oblique view >3 years after surgery.

A 17-year-old female presented with a rectangular left facial scar between the temporal and buccal region that was caused by a scald injury in childhood. The scar was 15×7.5 cm in size. The areas of normal skin at the sides of the scar were not sufficient for tissue expansion and transfer. Thus, an incision was performed inside the rectangular scar and two rectangular expanders were implanted under the cicatricial area and ambient normal skin. To ensure smooth healing of the incision, overlapping suturing of the dermal tissue was conducted. After 9 weeks of expander inflation, the facial scar was completely removed and the expanded flap was transferred to the center of the defect. More than three years after surgery, the incision scar was no longer evident and the patient was satisfied with the resulting appearance (Fig. 3).

#### Case 9

**Figure 4.**
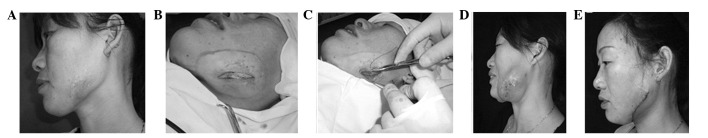
Case 9. Elimination of facial scar with expansion *in-situ* technique. (A) Preoperative view of a 30-year-old patient presented with a left facial scar. (B) De-epithelialization of the incision of implanting the expander in the operation. (C) The dermal flap on both sides of the incision and the overlapping staggered suture was made. (D) The lateral view after 8-week expansion *in-situ*; (E) the early postoperative view.

A 30-year-old female patient presented with a left facial scar caused by a burn in childhood. The scar was 6.0×3.0 cm. A 100-ml expander was implanted under the scar and overlapping suturing of the incision was conducted. The expanded flap was transferred to the center of the defect and the facial scar was completely removed after 8 weeks of expander inflation (Fig. 4).

## Discussion

The soft-tissue expansion technique was first reported in 1957 in a study by Neumann (9) in which a rubber balloon with an external port was used to reconstruct a traumatic ear defect. Since Radovan (10) reported breast reconstruction using tissue expansion in 1982, the clinical applications of soft-tissue expansion techniques have been continuously developed and used increasingly on a number of regions of the body. Traditional soft-tissue expansion methods use an expander that is gradually filled with saline to expand the overlying skin immediately adjacent to a wound. As this technique has been developed, the expander materials and application of the method have undergone various improvements (11–15). Currently, there are a wide array of expanders of different shapes and sizes that have been developed to maximize the volume of tissue expansion in any given anatomical location (16–18). Innovations, including the use of increasing numbers of tissue expanders and repeated expansion in the same region, have been achieved in difficult clinical situations (19–21). Thus, tissue expansion techniques are becoming an extremely important tool for plastic surgeons. Despite a number of benefits of tissue expansion in the reconstruction of large and complex soft-tissue defects, there are several significant problems that limit its clinical applicability. The drawbacks of traditional expansion techniques involve low utility rates of the expanded flap, additional incisions during flap transfer, prolonged expansion periods and high complication rates (4). These shortcomings are even more significant in the treatment of small defects of the face, neck and limbs that it is not possible to excise directly and suture in one procedure or via serial excision. Effective methods to repair these defects using tissue expansion techniques are not available. To solve these problems, certain improvements have been gradually applied and achieved during clinical situations.

**Figure 5.**
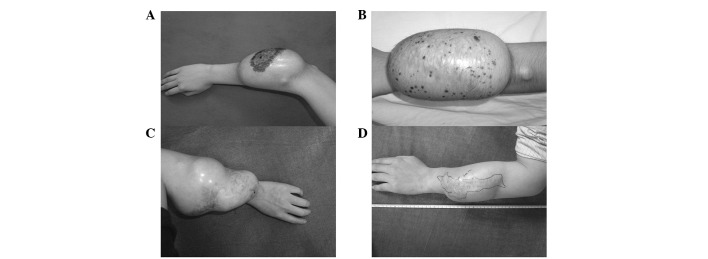
Expansion *in-situ* under various lesions. (A) Expansion under a giant nevus. (B) Expansion under scattered lesions. (C) Expansion under a tattoo of an irregular shape. (D) Expansion under a scar that was unable to be excised and closed directly.

Cai *et al* (22) were the first to report expansion under the cicatrix and the utilization rate and flexibility to transfer the expanded flap were shown to be increased. However, expansion was performed under the cicatrix only and the vertical incision made to implant the expander was in the normal area surrounding the cicatrix. In the present study, the tissue expander was implanted under the lesions besides the cicatrix (Fig. 5) and the dermal flaps created following the de-epithelialization of the lesion on both sides of the wound by insertion of the expander were closed by overlapping suturing. This method is named ‘expansion *in-situ*’. This technique used the lesion as the center and fully expanded the surrounding normal skin, so as to reduce the relative size of the lesion. In a two-stage operation, lesions were excised and the expanded flaps surrounding the defects were transferred to the center in order to close the wound directly without a great amount of tension. The flexibility of the expanded flap allowed an ‘S-shaped’ suture to be created. No additional incisions were made and no donor site morbidities occurred.

A number of techniques have also been devised in attempts to close the incision created for implantation of the expander (22). The overlapping suture aids the effective healing of dermal tissue and avoids wound dehiscence during the expansion period. The overlapping suture aids the effective healing of dermal tissue and avoids wound dehiscence during the expansion period. A partial subcutaneous scar may form in the overlapping area. The expansion capacity of cicatricial tissue is lower than that of the surrounding normal skin tissue. Hence, during expander inflation, the augmentation of surrounding normal skin tissue is greater than that of the overlapping section of lesions with the same expansion pressure. As a result, there is an expansion of normal skin tissue and relative reduction of the lesion area.

This method is not suitable for all lesions, including large lesions that it is not possible to resect via one-time expansion, lesions without sufficient surrounding normal skin tissue to implant an expander and lesions with a tendency for implantation metastasis. The method is more suitable for small- or medium-sized lesions that cannot be excised and sutured directly in a one-stage procedure or via serial excision. The expanded normal skin tissue that surrounds the lesion in all directions is fully utilized and allows closure of the defect by direct suturing without any tension. However, this technique may be combined with traditional expansion techniques in order to repair larger defects. The size of lesions suitable for resection using this method and the maximum bearing force of the overlapping suture area remain to be determined. Further studies are required to determine these factors. However, if these problems are solved, this technique is likely to be used more frequently in the future.

In conclusion, this method is suitable for repairing small- or medium-sized defects with normal surrounding skin tissue. It does not increase the risk of complications or the difficulty of surgery. Compared with traditional expansion techniques, the expansion *in-situ* technique implants an expander under the lesion, reducing the number of expanders required, the damaged caused and the treatment duration. This novel approach to expansion markedly improves the clinical usefulness of the expanded flap without the requirement for additional incisions or a risk of compromising the blood supply to the extended flaps. Certain factors, including the size of defects that it is possible to reconstruct using this method and the bearing force of the overlapping-suture area on the expansion, remain to be investigated.

## Figures and Tables

**Table I.** Summary of patient characteristics.

Case no.	Age (years)	Gender	Defect size (cm)	Cause	Expander volume (ml)	Inflation time (weeks)	Complications	Follow-up time (months)
1	16	F	3.0×2.5	Facial nevus	50	7	None	12
2	22	F	7.0×3.0	Facial scar	200	8	Poor incision healing	12
3	12	M	10.0×6.0	Upper limb nevus	300	10	None	12
4	27	F	3.0×2.5	Facial nevus	50	7	None	24
5	17	M	15.0×7.5	Facial scar	400 and 100	9	None	36
6	19	M	9.0×6.5	Upper limb nevus	200	8	None	None
7	17	M	9.0×5.0	Upper limb nevus	200	9	None	None
8	24	M	7.5×3.0	Upper limb scar	80	7	None	None
9	30	F	6.0×3.0	Facial scar	100	8	None	None
10	29	M	7.0×3.0	Upper limb scar	100	6	None	None

F, female; M, male.
